# Supplementary material for: Internet search analysis on the treatment of rheumatoid arthritis: What do people ask and read online?
Source: PLoS One. 2023 Sep 22;18(9):e0285869. doi: 10.1371/journal.pone.0285869 (PMC10516429; doi:10.1371/journal.pone.0285869)
Supplement: S1 Table — (DOCX) [file pone.0285869.s001.docx]

**S1 Table. Question classification (in Japanese, n = 83).**

| **Rothwell classification** | | **n (%)** | | **Example (in Japanese)** |
| --- | --- | --- | --- | --- |
|  | **Question sub-classification** | | **n (%)** |  |
| Fact | | 50 (60) | |  |
|  | General information | | 13 (16) | **リュウマチに効く薬はありますか？**  **リュウマチはどうしたら治りますか？** |
|  | Mechanism | | 13 (16) | **メトトレキサート / 葉酸 / 併用なぜ？**  **プレドニン / なぜ朝？** |
|  | Timeline of treatment | | 17 (20) | **リウマチの薬はいつまで続けるの？**  **リウマチ / 注射 / いつまで？** |
|  | Technical details | | 4 (5) | **リウマチ / 注射 / どこに打つ？** |
|  | Cost | | 3 (4) | **アダリムマブ / いくら？** |
| Policy | | 7 (8) | |  |
|  | Indication | | 0 (0) |  |
|  | Risk or complication | | 7 (8) | **メトトレキサート / 副作用 / いつ？** |
| Value | | 26 (31) | |  |
|  | Timeline of clinical course | | 13 (16) | **トシリズマブ / 効果 / いつから？**  **リウマチ / 関節破壊 / いつから？** |
|  | Prognosis | | 13 (16) | **関節リウマチは完治しますか？**  **リウマチ / 放置 / どうなる？** |
|  | Evaluation | | 0 (0) |  |
