# Supplementary material for: Internet search analysis on the treatment of rheumatoid arthritis: What do people ask and read online?
Source: PLoS One. 2023 Sep 22;18(9):e0285869. doi: 10.1371/journal.pone.0285869 (PMC10516429; doi:10.1371/journal.pone.0285869)
Supplement: S2 Table — (DOCX) [file pone.0285869.s002.docx]

**S2 Table. Domain scores of the website quality measurements (n = 83).**

|  | | Score | Possible score (worst–best) |
| --- | --- | --- | --- |
| Brief DISCERN | |  |  |
|  | Information sources (Q1) | 1 (1, 1) | 1–5 |
|  | Publication date (Q2) | 1 (1, 1) | 1–5 |
|  | How treatment works (Q3) | 2 (1, 3) | 1–5 |
|  | Benefits (Q4) | 2 (1, 3) | 1–5 |
|  | Risks (Q5) | 3 (2, 4) | 1–5 |
|  | Overall quality of life (Q6) | 1 (1, 1) | 1–5 |
| JAMA | |  |  |
|  | Authorship (Q1) | 0 (0, 1) | 0–1 |
|  | References (Q2) | 0 (0, 0) | 0–1 |
|  | Posting date and updates (Q3) | 0 (0, 0) | 0–1 |
|  | Disclosures (Q4) | 0 (0, 0) | 0–1 |
| CCI (raw score) | |  |  |
|  | Main message and call to action (Q1–5) | 0 (0, 1) | 0–5 |
|  | Language (Q6–7) | 1 (0, 1) | 0–2 |
|  | Information design ((8–10) | 2 (2, 2) | 0–3 |
|  | State of science (Q11) | 0 (0, 0) | 0–1 |

Values indicate the median (25^th^, 75^th^ percentiles). JAMA, Journal of the American Medical Association benchmark criteria; CCI, Clear Communication Index; Q, question. Note that the recommendation for action, numbers, and risk domains of the CCI (Q12-20) are not shown because they are optional questions.
