# Supplementary material for: Internet search analysis on the treatment of rheumatoid arthritis: What do people ask and read online?
Source: PLoS One. 2023 Sep 22;18(9):e0285869. doi: 10.1371/journal.pone.0285869 (PMC10516429; doi:10.1371/journal.pone.0285869)
Supplement: Attachment — Submitted filename: Response to Reviewers.docx [file pone.0285869.s004.docx]

We appreciate the thoughtful comments by the reviewers. All comments were incorporated into the revised manuscript. We hope that the revised version will be acceptable for publication.

Reviewer #1: 
[Reviewer comment]

As the authors point out, the fact that only Japanese is used is a limitation, but on the other hand, Japanese is a unique language in the world, used almost exclusively in Japan and as a native language by most Japanese people, thus the results of this study may represent a one-to-one correspondence with domestic trends in Japan. From this point of view, it would be possible to discuss the differences between the results in Japan and other countries.

[Author response]

We appreciate the reviewer's comment. We addressed the reviewer's point in the second paragraph of the discussion.

[Author action]

We added the following sentences.

"Another potential reason for the discrepancy between our study and the previous reports is that we searched Japanese words exclusively used domestically. Therefore, the results of this study may highlight the information needs of Japanese people and the difference between countries. Therefore, ..." (L231 of the revised manuscript)

[Reviewer comment]

The study was conducted in the midst of COVID-19 pandemic. It is reported that it was difficult to receive out-patient treatment for chronic diseases during COVID pandemic, and it is possible that patients sought information sources on the Internet. This point should be discussed.
[Author response]

We agree with the reviewer's comment. The reviewer's point was addressed in the limitation section. We also added a relevant reference to the manuscript.

[Author action]

We added the following sentence in the limitation section

"Finally, this study was conducted during the Coronavirus disease 2019 pandemic, when health information-seeking behavior on the internet might differ from that of the pre-pandemic period. Therefore, caution is needed to compare the results of this study with those of the literature." (L292 of the revised manuscript)

We added Zhang et al. as reference 31. (L418 of the revised manuscript)

[Reviewer comment]

As for the searching term, it would be appropriate to indicate the words actually inputted in kanji characters instead of romaji.
[Author response]

We revised the search terms according to the reviewer's comment.

[Author action]

The terms were revised as follows.

"The following six terms were searched: "rheumatoid arthritis" "treatment" ("関節リウマチ" "治療" in Japanese), "rheumatism"" treatment" ("リウマチ" "治療"), "rheumatoid arthritis" "pharmacotherapy" ("関節リウマチ" "薬物療法"), "rheumatism" "pharmacotherapy" ("リウマチ" "薬物療法"), "rheumatoid arthritis" "drugs" ("関節リウマチ" "薬"), and "rheumatism" "drugs" ("リウマチ" "薬")." (L99 of the revised manuscript)

[Reviewer comment]

Figure Legend should be added as it seems to be missing.
[Author response]

In the first submission, we did not add the legend of Fig. 1 because it is self-explanatory. However, according to the reviewer’s comment, we added the legend. We hope the revision will answer the reviewer's comment appropriately.

[Author action]

The following sentence was added.

“The top square indicates the initial website selection. The lower squares show the excluded websites and the numbers for analysis.” (L170 of the revised manuscript)

[Reviewer comment]

Please state the official name of the IRB which not requiring ethics review.
[Author response]

We added the official name of the IRB according to the reviewer's comment.

[Author action]

The IRB name was added as follows.

"…by the Research Ethics Committee of the Graduate School of Medicine, Chiba University because..." (L93 of the revised manuscript)

[Reviewer comment]

Please tell us the version of Google Chrome you used.
[Author response]

We added the information on the version.

[Author action]

We revised the sentence as follows.

“…Google Chromme browser (version 99.0.4844.51) to minimize…” (L98 of the revised manuscript)

[Reviewer comment]

Google may change the search results depending on where you accessed the Internet, so please share information such as place names and IP addresses rather than simply stating "Japan" if possible

[Author response]

We showed the city name and IP address according to the reviewer's comment.

[Author action]

We made the following revision.

"...April 21, 2022, in Chiba City, Japan (internet protocol address, 10.106.102.246) using…" (L96 of the revised manuscript)

Reviewer #2: 
[Reviewer comment]

Regarding the terms such as ''Single surgeon personal'', ''Websites maintained by individual surgeons without an institutional affiliation'' especially in Table 3, I know some area orthopedic surgeon is treating rheumatoid arthritis. However, those patients are usually treated by rheumatologist(or physician) in general all over the world. Therefore, this term may need to be changed to other phrase. For instance, single physician or surgeon personal or single medical doctor, which include physicians and surgeon.

[Author response]

We agree with the reviewer's comment. The term was revised to single medical doctor.

[Author action]

We revised the manuscript as follows.

Table 1.

| Single medical doctor | Websites maintained by individual medical doctors without an institutional affiliation |
| --- | --- |

"...medical practice, single medical doctor, and..." (L133 of the revised manuscript)

"... Government and single medical doctor websites were..." (L188 of the revised manuscript)

[Reviewer comment]

Regarding in Table 2, there was a word ''Humira''. It should be changed to generic name, Adalimumab''.
[Author response]

The word "Humira" was used because we directly traced the questions that appeared on "People Also Ask." However, we rephrased the word according to the reviewer's comment. We also rephrased Actemura to tocilizumab

[Author action]

We made the following revision.

Table 2.

| Cost | 3 (4) | Adalimumab / How much? | |
| --- | --- | --- | --- |
| Policy | 7 (8) | |  |
| Indication | 0 (0) |  | |
| Risk or complication | 7 (8) | Methotrexate / Side effects / From when? | |
| Value | 26 (31) | |  |
| Timeline of clinical course | 13 (16) | Tocilizumab / Effect / From when?  Rheumatism / Joint destruction / From when? | |

Supporting information

| Cost | 3 (4) | **アダリムマブ / いくら？** | |
| --- | --- | --- | --- |
| Policy | 7 (8) | |  |
| Indication | 0 (0) |  | |
| Risk or complication | 7 (8) | **メトトレキサート / 副作用 / いつ？** | |
| Value | 26 (31) | |  |
| Timeline of clinical course | 13 (16) | **トシリズマブ / 効果 / いつから？**  **リウマチ / 関節破壊 / いつから？** | |

[Reviewer comment]

Lastly, for all the tables, probably it can be sorted clearly so subscribers understand better.

[Author response]

We revised the tables (including supporting information) and hope that the revision will address the reviewer’s concern. We will be happy to revise further if the reviewer suggests specific points.

[Author action]

We revised the tables as follows.

Table 1.

| **Rothwell classification** | | | **Description** |
| --- | --- | --- | --- |
| Fact | | | Asks objective, factual information |
| Policy | | | Asks information on a specific course of action that should be taken to solve a problem |
| Value | | | Asks for evaluation of an idea, object, or event |
|  | | | |
| **Question subclassification** | | | **Description** |
| Fact | |  | |
|  | General information | | General questions not categorized below |
|  | Mechanism | | Mechanism of treatment |
|  | Timeline of treatment | | Questions regarding the length of time for treatment |
|  | Technical details | | Treatment procedures on medications |
|  | Cost | | Cost of treatment |
| Policy | |  | |
|  | Indication | | Indication of treatment |
|  | Risk or complication | | Risks or complications during treatment |
| Value | |  | |
|  | Timeline of clinical course | | Questions regarding the length of time for clinical course |
|  | Prognosis | | Questions regarding the consequence of treatment |
|  | Evaluation | | Evaluation of treatment |
|  | | | |
| **Answer source website categorization** | | | **Description** |
| Academic | | | Academic institutions, including universities, academic medical centers, and academic societies |
| Commercial | | | Commercial organizations, such as medical device and pharmaceutical companies |
| Government | | | Websites ending in “.gov” or maintained by government organizations |
| Medical practice | | | Local hospitals or orthopedic practices without an academic affiliation |
| Single medical doctor | | | Websites maintained by individual medical doctors without an institutional affiliation |
| Social media | | | Nonmedical organizations and websites intended for information sharing |

Table 2.

| **Rothwell classification** | | **n (%)** | | **Example** |
| --- | --- | --- | --- | --- |
|  | **Question subclassification** | | **n (%)** |  |
| Fact | | 50 (60) | |  |
|  | General information | | 13 (16) | Are there any medications that work for rheumatism?  How can rheumatism be treated? |
|  | Mechanism | | 13 (16) | Methotrexate / Folic acid / Why combined?  Prednisone / Why morning? |
|  | Timeline of treatment | | 17 (20) | Until when do I continue rheumatoid medications?  Rheumatism / Injection / Until when? |
|  | Technical details | | 4 (5) | Rheumatism / Injection / Where? |
|  | Cost | | 3 (4) | Adalimumab / How much? |
| Policy | | 7 (8) | |  |
|  | Indication | | 0 (0) |  |
|  | Risk or complication | | 7 (8) | Methotrexate / Side effects / From when? |
| Value | | 26 (31) | |  |
|  | Timeline of clinical course | | 13 (16) | Tocilizumab / Effect / From when?  Rheumatism / Joint destruction / From when? |
|  | Prognosis | | 13 (16) | Can rheumatoid arthritis be cured?  Rheumatism / Leave untreated / What happens? |
|  | Evaluation | | 0 (0) |  |

Table 3.

|  | **Overall**  **(n = 83)** | **Academic**  **(n = 17)** | **Commercial**  **(n = 8)** | **Medical practice**  **(n = 46)** | **Social media**  **(n = 12)** | ***P*** |
| --- | --- | --- | --- | --- | --- | --- |
|  | | | | | | |
| Brief DISCERN | 11 (8, 13) | 12 (10, 13) | 14 (11, 14) | 9 (8, 12) | 17 (9, 19) | < 0.001 |
|  | | | | | | |
| JAMA | 0 (0, 1) | 0 (0, 1) | 1 (1, 1) | 0 (0, 1) | 1 (1, 1) | < 0.001 |
|  | | | | | | |
| CCI | 40 (40, 47) | 40 (30, 52) | 40 (27, 40) | 42 (40, 47) | 49 (43, 50) | 0.01 |

Table 4.

|  | **Question subclassification**^a^ | **Website category**^a^ | **Brief DISCERN**^b^ | **JAMA**^b^ | **CCI**^b^ |
| --- | --- | --- | --- | --- | --- |
| Intrarater | 0.94 (0.89, 0.99) | 1.00 | 0.94 (0.85, 0.97) | 0.89 (0.76, 0.96) | 0.87 (0.71, 0.95) |
| Interrater | 0.75 (0.63, 0.84) | 0.86 (0.67, 1.00) | 0.92 (0.81, 0.97) | 0.94 (0.87, 0.98) | 0.87 (0.71, 0.95) |

Supporting information

| **Rothwell classification** | | **n (%)** | | **Example (in Japanese)** |
| --- | --- | --- | --- | --- |
|  | **Question subclassification** | | **n (%)** |  |
| Fact | | 50 (60) | |  |
|  | General information | | 13 (16) | **リュウマチに効く薬はありますか？**  **リュウマチはどうしたら治りますか？** |
|  | Mechanism | | 13 (16) | **メトトレキサート / 葉酸 / 併用なぜ？**  **プレドニン / なぜ朝？** |
|  | Timeline of treatment | | 17 (20) | **リウマチの薬はいつまで続けるの？**  **リウマチ / 注射 / いつまで？** |
|  | Technical details | | 4 (5) | **リウマチ / 注射 / どこに打つ？** |
|  | Cost | | 3 (4) | **アダリムマブ / いくら？** |
| Policy | | 7 (8) | |  |
|  | Indication | | 0 (0) |  |
|  | Risk or complication | | 7 (8) | **メトトレキサート / 副作用 / いつ？** |
| Value | | 26 (31) | |  |
|  | Timeline of clinical course | | 13 (16) | **トシリズマブ / 効果 / いつから？**  **リウマチ / 関節破壊 / いつから？** |
|  | Prognosis | | 13 (16) | **関節リウマチは完治しますか？**  **リウマチ / 放置 / どうなる？** |
|  | Evaluation | | 0 (0) |  |

Other corrections

The following sentence was added to the manuscript for additional information of the data.

“All relevant data are within the manuscript and its Supporting Information files. This study complies with the Declaration of Helsinki.” (L94 of the revised manuscript)
