# Supplementary material for: Internet search analysis on the treatment of rheumatoid arthritis: What do people ask and read online?
Source: PLoS One. 2023 Sep 22;18(9):e0285869. doi: 10.1371/journal.pone.0285869 (PMC10516429; doi:10.1371/journal.pone.0285869)
Supplement: Attachment — Submitted filename: Response to Reviewers.docx [file pone.0285869.s005.docx]

Reviewer #1:

[Reviewer comment]

The manuscript is well addressed, however, the manuscript should be proofread by a native English speaker.

[Author response]

Thank you for the reviewer's comment. We assume the reviewer's comment is on the revised part because the English issue was not raised in the first submission. Although a professional English editor has proofread the submitted manuscript, we again corrected the revised and original parts. We hope that the manuscript will be acceptable for publication.

[Author action]

The following corrections were made.

"Furthermore, we searched Japanese words that were exclusively used domestically. Therefore, the results of this study highlighted the information needs of Japanese people, which may differ from those of people in other countries." (L229 of the revised manuscript)

Finally, this study was conducted during the Coronavirus disease 2019 pandemic, when health information-seeking behavior on the internet might differ from that of the pre-pandemic period. Therefore, a comparison between the results of this study and previous studies should be made with caution. (L299 of the revised manuscript)

Additionally, we corrected the manuscript as follows.

“The median score of brief DISCERN…” (L33 of the revised manuscript)

“…a vital part of the management of...” (L43 of the revised manuscript)

“…changing dramatically in the past decade” (L51 of the revised manuscript)

“…we can retrieve the most commonly…” (L73 of the revised manuscript)

“…has not yet been evaluated. (L84 of the revised manuscript)

“…that used a similar methodology.” (L108 of the revised manuscript)

“…After the extraction of data” (L110 of the revised manuscript)

“…into six groups.” (L110 of the revised manuscript)

“…comprises six questions…” (L110 of the revised manuscript)

“…having sufficient quality…” (L188 of the revised manuscript)

“…Japanese people frequently searched for…” (L213 of the revised manuscript)

“…the public's information needs regarding…” (L217 of the revised manuscript)

“…promoting adherence to treatment is challenging” (L238 of the revised manuscript)

“…he website category and quality is conflicting ..” (L268 of the revised manuscript)

“…most of the questions…” (L302 of the revised manuscript)

We corrected the brackets in the Abstract to avoid double brackets.

“(n = 17, 23%) and …” (L32 of the revised manuscript)

“…(n = 13, 16%).” (L33 of the revised manuscript)

“…(n = 17, 20%, Table 2 and supplementary material) (L174 of the revised manuscript)

“…(n =46, 55%, Table 3).” (L185 of the revised manuscript)

Reviewer #3

[Reviewer comment]
- This PAA system reflects the tendency of questions of what time period? Today? For the last 1 week? For the last one month?Does it reflect the general tendency of questions correctly or is there a possibility that the results may be affected by one temporary "phase" of search which is affected by a recent popular TV program for example? I think if the data sampling period is too short, there is the risk of misinterpreting a random rise in the search number as a general trend.

[Author response]

We appreciate the reviewer's thoughtful comment. The data used by the PAA algorithm is the range of the most recent information related to the keywords searched. The range of data varies by search term and is not limited to a specific time period. Therefore, it is unknown what period of internet information was used in the search for this specific study. We mentioned the reviewer's point in the limitation section.

[Author action]

We added the following sentences.

"...it is unknown what period of internet information was used to generate the PAA search results presented by Google. Furthermore, the results may change..." (L280 of the revised manuscript)

[Reviewer comment]
- I assume google search system tries to direct viewers to a website that gives quick and easy answer to each question. From that perspective, the websites that were linked to PAA questions do not necessarily have to be detailed, high quality or thorough regarding the general topic such as "treatment in general". You concluded that the quality of websites on RA treatment was insufficient. However, from this study, this big conclusion cannot be obtained because you did not collect the websites that explains "RA treatment in general." You assessed websites that "answered each commonly asked question" and assessed their quality. If you want to argue that "websites on RA treatment has poor quality", you would have to directly go to the websites that pop up with the general search "rheumatoid arthritis/treatment" etc, not the website that were linked with PAA. Once again, the websites that you picked up by PAA are the ones that likely give people "easy answers." for each small question.

[Author response]

We agree with the reviewer's comment that we assessed websites that answered each commonly asked question and did not assess websites in general. We revised the conclusion sentences in the Abstract and main text according to the reviewer's comment.

We did not assess the general search result but assessed the websites linked to PAA. This is because the main purpose of this study was to clarify the patient's needs using the PAA questions.

[Author action]

The following sentence in the Abstract was deleted.

"The quality of websites about RA treatment was insufficient." (L39 of the previous submission)

The sentence in the conclusion paragraph was revised as follows.

"…the quality of the answer source websites was insufficient." (L304 of the revised manuscript)

[Reviewer comment]
- I think there is weakness of this search method. This study is based on the premise that people start searching with specific 6 combinations of terms. However, this is not always true in the real world. For example, if people searched with more specific terms such as "rheumatoid arthritis, biologics", worrying about the high cost of biologics, that tendency would not be reflected on the result of this study. Others may search with "rheumatoid arthritis, side effects" or "rheumatoid arthritis, methotrexate" "rheumatoid arthritis, infectious risk". Many patients often have specific concerns and they tend to search with more specific terms depending on their concerns.
Therefore, this study dose shed light to one aspect of people's concerns affected by random bias from the search engine but does not reflect the real-life frequency of the general population. Actual interview style studies from a smaller RA cohort would have less bias, reflecting the true tendency of question.

[Author response]

"This study is based on the premise that people start searching with specific 6 combinations of terms. However, this is not always true in the real world."

→ We agree with the reviewer's comment that the search results depend on the search terms. The reviewer's point was addressed in the limitation section.

"Many patients often have specific concerns and they tend to search with more specific terms depending on their concerns."

"…but does not reflect the real-life frequency of the general population"

→ The reviewer's comment may be correct, although we did not find information that supports the reviewer's comment. However, most internet search studies used general terms so that the results can be generalized to a broad population. The search results from the more specific terms would yield more specific patient needs but apply to a limited patient group. Therefore, either approach (general or specific) has advantages and disadvantages. The reviewer's point was addressed in the limitation section.

"Actual interview style studies from a smaller RA cohort would have less bias, reflecting the true tendency of question."

→ A comparison between the internet search and interview is beyond the topic of this study because we did not conduct an interview. Furthermore, both the internet search and interview studies have specific biases. Therefore, it would be difficult to determine whether the interview has less bias than the internet search.

Nevertheless, we are willing to incorporate the reviewer's point into the revised manuscript. We could not find information and research that support the reviewer's comment "interview style studies from a smaller RA cohort would have less bias than internet search studies, reflecting the true tendency of question." Could the reviewer kindly provide the relevant information?

[Author action]

The following sentences were added to the limitation section.

"Fifth, Internet search results may vary depending on the search terms. We searched for general terms such as "treatment," similar to the previous studies [8, 12, 13, 18]. However, searching for more specific words, such as "biologics," may reveal more specific patient needs at the cost of limited generalizability." (L293 of the revised manuscript)

Again, we will further revise the manuscript if the reviewer could kindly provide information that an interview style is less biased than an internet search style.

[Reviewer comment]
- Are there any websites that were rated as "good" in Japan in general? Even academic websites that were linked failed to meet "good quality" criteria. Can this be a problem of the outcome measures that were used? Or can this be a misguidance by google to a low quality website? Or does this reflect poor quality of Japanese online resources in general?
If you think there might be a problem with the quality measures, I would want to hear more about why and in what way. (More details in the next section.) If you think it could be a google misguidance problem despite the existence of high quality websites in Japan, you might want to mention examples of websites with "good quality" in Japan. If you think this is because of the general paucity of such websites in Japan (although I do not believe this study can directly support this conclusion), you might want to elaborate on that.

[Author response]

We appreciate the reviewer's comment.

"Are there any websites that were rated as "good" in Japan in general?"

→ As shown in the Results, 8% of the websites were rated as having sufficient quality based on DISCERN.

"Or does this reflect poor quality of Japanese online resources in general?"

→ As we already mentioned in the Discussion (L251 of the revised manuscript), the results of this study may reflect the poor quality of Japanese online resources in general. This is because the previous studies consistently reported insufficient quality of Japanese websites (ref. 19, 20, 27, and Yaguchi-Saito et al. Drug Discoveries ＆ Therapeutics. 2021). However, as already mentioned in the Discussion, we would like to highlight that insufficient quality of healthcare websites was prevalent not only in Japan but also throughout the world (L255 of the revised manuscript).

"If you think this is because of the general paucity of such websites in Japan (although I do not believe this study can directly support this conclusion), you might want to elaborate on that."

→ According to the reviewer's comment, we further elaborate on this issue.

[Author action]

The following sentences were added to the Discussion.

"Furthermore, the assessment tools in this study can be used to improve the quality of patient information [29, 30]." (L262 of the revised manuscript)

The following references were added.

29. Porter KJ, Alexander R, Perzynski KM, Kruzliakova N, Zoellner JM. Using the Clear Communication Index to Improve Materials for a Behavioral Intervention. Health Commun. 2019;34:782-788. https://doi.org/10.1080/10410236.2018.1436383 (L411 of the revised manuscript)

30. Centers for Disease Control and Prevention. The CDC Clear Communication Index. Example Materials. [cited 2023 Apr 8]. Available from: <https://www.cdc.gov/ccindex/examplematerial/index.html> (L414 of the revised manuscript)

The reference numbers have been changed accordingly.

Ref. 29→31 (L417 of the revised manuscript)

Ref. 30→32 (L421 of the revised manuscript)

Ref. 31→33 (L425 of the revised manuscript)

[Reviewer comment]
- Regarding the validity of the quality measures, it concerns me that social media is rated as the highest quality compared with academic or medical practice websites because it is contraty to my intuition. I would imagine academic entities would provide more accurate, less biased and updated information than social media, which has no guarantee regarding bias or background knowledge of the authors. You did mention other studies using the same quality measures in the discussion. Do you think these measures appropriately evaluated the quality of RA websites? Do you think that academic website has information with lower quality than social media? If you think there is some problem with these quality measures, you should comment on that in the discussion because I think these questions are crucial to this paper. The reliability of outcome depends on the validity of these quality measures.

[Author response]

"I would imagine academic entities would provide more accurate, less biased and updated information than social media"

"Do you think these measures appropriately evaluated the quality of RA websites?"

"If you think there is some problem with these quality measures, you should comment on that in the discussion because I think these questions are crucial to this paper. The reliability of outcome depends on the validity of these quality measures."

→ As already mentioned in the paper (L132 of the revised manuscript), these measures have been validated and extensively used for the quality assessment of healthcare websites, including RA websites. Therefore, we believe these measures appropriately evaluated the RA websites. We stated that the tools have been validated.

However, these measures do not evaluate the accuracy/correctness of the information. Furthermore, they evaluated only the website update and do not assess whether the content of information was truly updated. The information correctness is another metric than the quality and beyond the scope of this study. We addressed this issue in the Discussion. We hope that the reviewer will kindly accept our response.

"social media is rated as the highest quality compared with academic or medical practice websites"

"Do you think that academic website has information with lower quality than social media?

→Please note that the scores of academic websites were *not* lower than those of social media websites as shown in Table 3. Our results showed that the quality of *medica*l practice websites was statistically lower than that of social media websites.

As mentioned in L271, a previous study (ref. 29) reported that academic website had low quality. Therefore, academic websites may be always high-quality based on the criteria used in this study. However, statistical significance does not necessarily mean clinical significance. Furthermore, we did not intend to highlight the low quality in medical practice websites but intended to emphasize overall low quality regardless of the category.

[Author action]

We revised the sentence in the Methods.

“The quality of the websites was evaluated using validated tools:...” (L132 of the revised manuscript)

We added the following sentences in the Discussion.

"Furthermore, the evaluation tools used in this study did not assess the correctness of the information. Therefore, even websites rated as high quality may contain incorrect information." (L275 of the revised manuscript)

[Reviewer comment]

Overall, I liked this novel attempt in filling the knowledge gap between patients and physicians. I do think your messages here at least partially hold truth from personal clinical experience. However, I also think a few questions regarding logics and the design that were mentioned above should be addressed.

[Author response]

We appreciate the reviewe’s detailed comments. We acknowledge the limitation of the methodology and incorporate the reviewer’s comments into the revised manuscript.

[Other corrections]

Font of the words in Table 1, 2, 3, and 4, were corrected.

We added ref. 16 and 33 to the text that were missing. We apologize for our mistake.

"… public communication products [16]. (L143 of the revised manuscript)

"… the pre-pandemic period [33]." (L299 of the revised manuscript)
