# Supplementary material for: Internet search analysis on the treatment of rheumatoid arthritis: What do people ask and read online?
Source: PLoS One. 2023 Sep 22;18(9):e0285869. doi: 10.1371/journal.pone.0285869 (PMC10516429; doi:10.1371/journal.pone.0285869)
Supplement: Attachment — Submitted filename: Response to Reviewers.docx [file pone.0285869.s006.docx]

Reviewer #3:

[Reviewer comment]

I have one more request regarding the second conclusion of the study. I would like to hear a little more details regarding the low quality of websites. I would like to see what elements are lacking. You state that websites should have better quality. You must have assessed the websites based on 3 scoring systems and you should know which part websites scored less than ideal. It would be better to show what improvements are to be made than to just say they have low quality.

[Author response]

We appreciate the reviewer’s comment. According to the reviewer’s comment, we added the domain scores of website quality measurements as a supplementary table. We also mentioned the results in the manuscript.

[Author action]

The following sentences were added to the Results and Discussion.

“Regarding the domain scores of the measurements, those on information transparency, such as the Brief DISCERN questions 1 and 2, all JAMA benchmark criteria questions, and CCI question 11, were low (S2). Additionally, scores were low on questions related to the overall quality of life (brief DISCERN question 1) and main message and call to action (CCI questions 1–5). However, the scores of the other domains were also not sufficient.” (L191-196 of the revised manuscript)

Especially, descriptions of information transparency were lacking. (L311-312 of the revised manuscript)

We added the supplementary figure.

**S2 table. Domain scores of the website quality measurements (n = 83).**

|  | | Score | Possible score (worst–best) |
| --- | --- | --- | --- |
| Brief DISCERN | |  |  |
|  | Information sources (Q1) | 1 (1, 1) | 1–5 |
|  | Publication date (Q2) | 1 (1, 1) | 1–5 |
|  | How treatment works (Q3) | 2 (1, 3) | 1–5 |
|  | Benefits (Q4) | 2 (1, 3) | 1–5 |
|  | Risks (Q5) | 3 (2, 4) | 1–5 |
|  | Overall quality of life (Q6) | 1 (1, 1) | 1–5 |
| JAMA | |  |  |
|  | Authorship (Q1) | 0 (0, 1) | 0–1 |
|  | References (Q2) | 0 (0, 0) | 0–1 |
|  | Posting date and updates (Q3) | 0 (0, 0) | 0–1 |
|  | Disclosures (Q4) | 0 (0, 0) | 0–1 |
| CCI (raw score) | |  |  |
|  | Main message and call to action (Q1–5) | 0 (0, 1) | 0–5 |
|  | Language (Q6–7) | 1 (0, 1) | 0–2 |
|  | Information design ((8–10) | 2 (2, 2) | 0–3 |
|  | State of science (Q11) | 0 (0, 0) | 0–1 |

Values indicate the median (25^th^, 75^th^ percentiles). JAMA, Journal of the American Medical Association benchmark criteria; CCI, Clear Communication Index; Q, question. Note that the recommendation for action, numbers, and risk domains of the CCI (Q12-20) are not shown because they are optional questions.

**“S2 table. Domain scores of the website quality measurements (n = 83).”** (L439 of the revised manuscript)

Accordingly, the supplementary data in the previous manuscript was renamed as S1.

“...(n = 17, 20%, Table 2 and S1). “(L174 of the revised manuscript)
